# Echinohalimane A, a Bioactive Halimane-Type Diterpenoid from a Formosan Gorgonian *Echinomuricea* sp. (Plexauridae)

**DOI:** 10.3390/md10102246

**Published:** 2012-10-17

**Authors:** Hsu-Ming Chung, Li-Chung Hu, Wei-Hsuan Yen, Jui-Hsin Su, Mei-Chin Lu, Tsong-Long Hwang, Wei-Hsien Wang, Ping-Jyun Sung

**Affiliations:** 1 Department of Marine Biotechnology and Resources and Asia-Pacific Ocean Research Center, National Sun Yat-sen University, Kaohsiung 804, Taiwan; Email: shiuanmin@yahoo.com.tw (H.-M.C.); x2219@nmmba.gov.tw (J.-H.S.); 2 National Museum of Marine Biology and Aquarium, Pingtung 944, Taiwan; Email: stoja582@gmail.com (L.-C.H.); xyz78714@hotmail.com (W.-H.Y.); jinx6609@nmmba.gov.tw (M.-C.L.); 3 Graduate Institute of Marine Biotechnology and Department of Life Science and Institute of Biotechnology, National Dong Hwa University, Pingtung 944, Taiwan; 4 Graduate Institute of Natural Products, Chang Gung University, Taoyuan 333, Taiwan; Email: htl@mail.cgu.edu.tw

**Keywords:** halimane, echinohalimane, *Echinomuricea*, cytotoxicity, elastase

## Abstract

A new halimane-type diterpenoid, echinohalimane A (**1**), was isolated from a gorgonian, identified as *Echinomuricea* sp. The structure of **1** was determined by spectroscopic methods and this compound was found to exhibit cytotoxicity toward various tumor cells and display an inhibitory effect on the release of elastase by human neutrophils. Echinohalimane A (**1**) is the first halimane analogue from the marine organisms belonging to phylum Cnidaria.

## 1. Introduction

**Figure 1:**
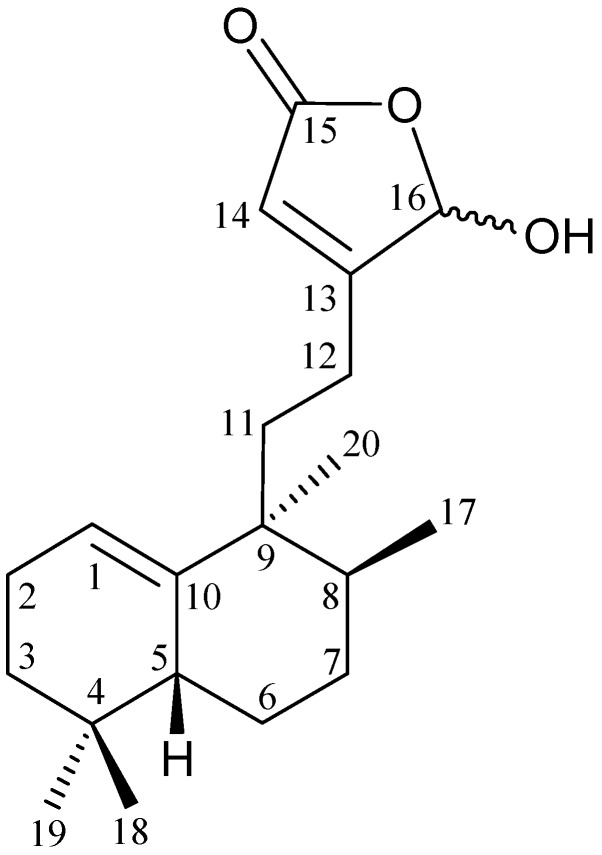
The structure of echinohalimane A (**1**).

The search for bioactive natural products from marine organisms has been remarkably successful [1] and octocorals have proven to be rich sources of interesting natural products [2,3]. In continuation of our search for new natural products from the marine invertebrates collected off the waters of Taiwan at the intersection of the Kuroshio current and the South China Sea surface current, a new bioactive substance, echinohalimane A (**1**) (Figure 1), was isolated from the gorgonian *Echinomuricea* sp. In this paper, we describe the isolation, structure determination and biological activities of echinohalimane A (**1**).

## 2. Results and Discussion

In previous studies, two bisabolane-type sesquiterpenoids, (7*S*,10*R*)-(+)-10,11-epoxycurcuphenol and (+)-curcuphenol [4]; a labdane-type diterpenoid, echinolabdane A [5]; a clerodane-type diterpenoid, echinoclerodane A [6]; and a steroid analogue, 6-*epi*-yonarasterol B [5], had been isolated from a Formosan gorgonian coral identified as *Echinomuricea* sp. (Plexauridae). We have further isolated a new halimane-type diterpenoid, echinohalimane A (**1**), from *Echinomuricea* sp.

Echinohalimane A (**1**) was isolated as a yellowish oil that gave a pseudomolecular ion (M + Na)^+^ at *m/z* 341.2089 in the HRESIMS, indicating the molecular formula C_20_H_30_O_3_ (calcd for C_20_H_30_O_3_ + Na, 341.2093) and implying six degrees of unsaturation. IR absorptions were observed at 3375 and 1755 cm^−1^, suggesting the presence of hydroxy and ester groups in **1**. The ^13^C NMR for **1** confirmed the presence of 20 carbon signals (Table 1), which were characterized by DEPT as four methyls, six sp^3^ methylenes, three sp^3^ methines, two sp^2^ methines, two sp^3^ quaternary carbons and three sp^2^ quaternary carbons. A suite of resonances at *δ*_C_ 172.2 (C-15), 171.6 (C-13), 116.7 (CH-14) and 99.4 (CH-16), could be assigned to an α,β-unsaturated-γ-lactone moiety [6]. A trisubstituted olefin was deduced from the ^13^C NMR data at *δ*_C_ 145.0 (C-10) and 118.1 (CH-1). Thus, from the reported data, the proposed skeleton of **1** was suggested to be a diterpenoid with three rings. 

**Table 1:** ^1^H (400 MHz, CDCl_3_) and ^13^C (100 MHz, CDCl_3_) NMR data, ^1^H–^1^H COSY and HMBC correlations for diterpenoid **1**.

Position	*δ*_H_ (*J* in Hz)	*δ*_C_, Multiple	^1^H–^1^H COSY	HMBC
1	5.40 br s	118.1, CH	H_2_-2	C-2, -9
2	2.02 m	23.2, CH_2_	H-1, H_2_-3	C-1, -3, -4, -10
3	1.11 m, 1.28 m	31.2, CH_2_	H_2_-2	C-2, -4, -18, -19
4		31.2, C		
5	1.49 br d (14.8)	43.7, CH	H_2_-6	C-3, -4, -7, -10
6	1.09 m, 1.80 m	30.0, CH_2_	H-5, H_2_-7	n.o.
7	1.42 m, 1.55 m	31.1, CH_2_	H_2_-6, H-8	C-8, -9
8	1.32 m	44.3, CH	H_2_-7, H_3_-17	C-7, -9, -10, -17
9		42.5, C		
10		145.0, C		
11	1.32 m, 1.90 m	28.2, CH_2_	H_2_-12	C-10, -13, -20
12	2.10 m	22.3, CH_2_	H_2_-11	
13		171.6, C		
14	5.79 s	116.7, CH		C-13, -15, -16
15		172.2, C		
16	5.98 s	99.4, CH		C-13, -14, -15
17	0.86 d (6.4)	16.3, CH_3_	H-8	C-7, -8, -9
18	0.85 s	27.5, CH_3_		C-3, -4, -5, -19
19	0.84 s	27.7, CH_3_		C-3, -4, -5, -18
20	1.04 s	22.9, CH_3_		C-8, -9, -10, -11

n.o. = not observed.

**Figure 2:**
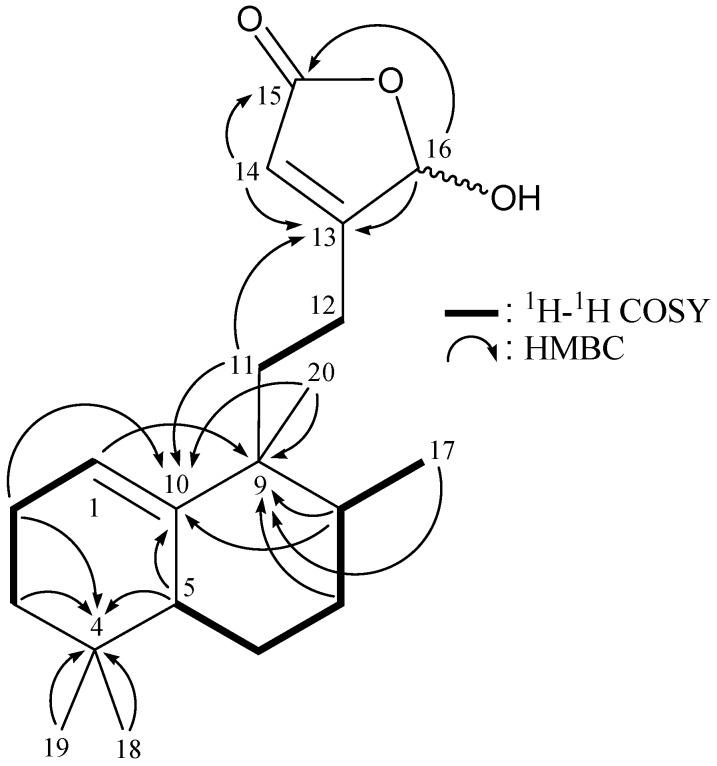
The ^1^H–^1^H COSY and selective HMBC correlations (protons→quaternary carbons) for diterpenoid **1**.

From the ^1^H–^1^H COSY experiment of **1** (Table 1 and Figure 2), it was possible to establish the spin systems that map out the proton sequences from H-1/H_2_-2/H_2_-3, H-5/H_2_-6/H_2_-7/H-8/H_3_-17 and H_2_-11/H_2_-12, which was accomplished with the assistance of an HMBC experiment (Table 1 and Figure 2). The key HMBC correlations between protons and quaternary carbons of **1**, including H_2_-2, H_2_-3, H-5, H_3_-18, H_3_-19/C-4; H-1, H_2_-7, H-8, H_3_-17, H_3_-20/C-9; H_2_-2, H-5, H-8, H_2_-11, H_3_-20/C-10; H_2_-11, H-14, H-16/C-13; and H-14, H-16/C-15, permitted the elucidation of the carbon skeleton of **1**. The tertiary methyls at C-4, C-8 and C-9 were confirmed by the HMBC correlations between H_3_-18/C-3, -4, -5, -19; H_3_-19/C-3, -4, -5, -18; H_3_-17/C-7, -8, -9; and H_3_-20/C-8, -9, -10, -11. The methine unit at *δ*_C_ 99.4 (CH-16) was more shielded than that expected for an oxygenated C-atom and correlated with the methine proton at *δ*_H_ 5.98 (H-16) in the HMQC spectrum, and this proton showed ^3^*J*-correlations with C-14 and C-15 in the HMBC spectrum and concluded to be a part of a hemiketal constellation.

**Figure 3:**
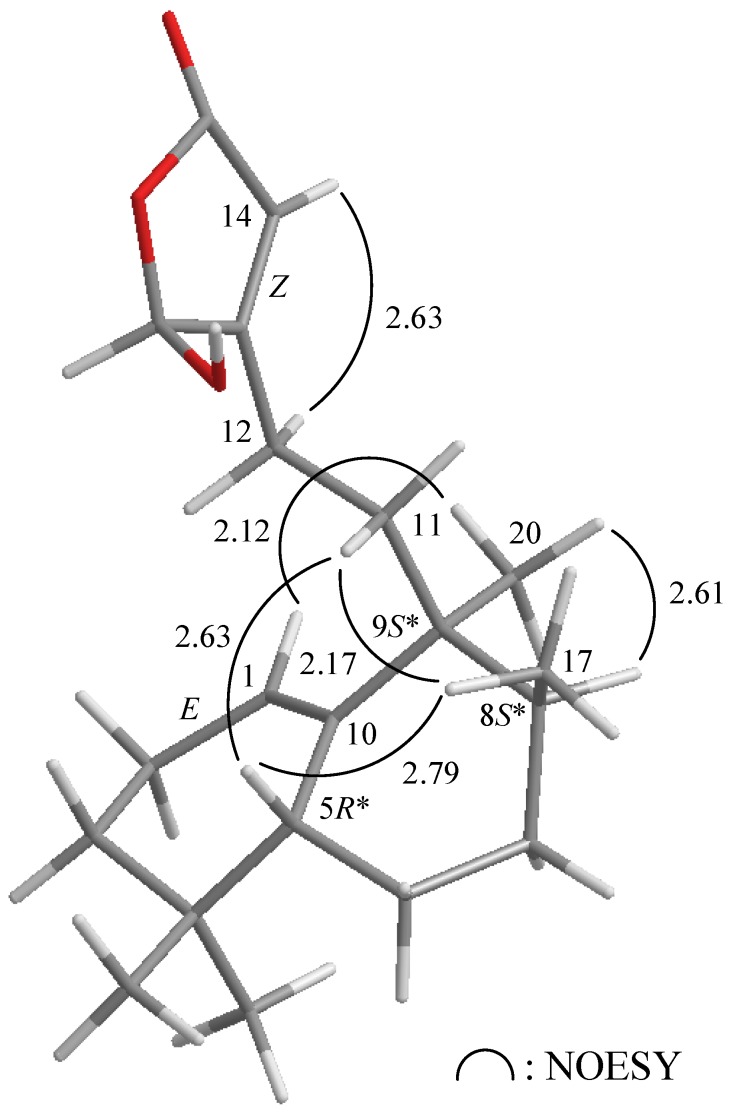
The stereoview of **1** (generated from computer modeling) and the calculated distances (Å) between selected protons having key NOESY correlations.

The relative configuration of **1** was elucidated mainly from a NOESY spectrum as being compatible with that of **1** ascertained using molecular mechanics calculations (MM2), which suggested the most stable conformations, as shown in Figure 3 [7], in which the close contacts of atoms in space calculated were consistent the NOESY correlations. In the NOESY experiment of **1**, H-5 exhibited correlations with H_3_-17 and a proton of C-11 methylene (*δ*_H_ 1.90), but not with H-8 and H_3_-20, indicated that H-5 and Me-17 were situated on the same face in **1**, and these were assigned as β protons, since the C-20 methyl is an α-substituent at C-9. The *E-*configuration of C-1/10 double bond was elucidated from a correlation between H-1 and H_3_-20. Based on the above findings, the main structure of **1** was elucidated unambiguously, and the chiarl carbons for **1** were assigned as 5R*, 8S*, and 9S* although the configuration for 16-hydroxy group was not determined at this stage by this method.

The cytotoxicity of diterpenoid **1** toward K562 (human erythromyeloblastoid leukemia), MOLT-4 (human acute lymphoblastic leukemia), HL-60 (human acute promyelocytic leukemia), DLD-1 (human colorectal adenocarcinoma), LoVo (human colorectal adenocarcinoma) and DU-145 (human prostate carcinoma) cells was studied, and the results were shown in Table 2. These data showed that echinohalimane A (**1**) exhibited cytotoxicity toward MOLT-4, HL-60, DLD-1 and LoVo cells. Furthermore, the *in vitro* anti-inflammatory effects of diterpenoid **1** were tested and echinohalimane A (**1**) displayed a significant inhibitory effect on the release of elastase by human neutrophils (Table 3).

**Table 2:** Cytotoxic data of diterpenoid **1**.

Compounds	Cell lines IC_50_ (μg/mL)					
	K562	MOLT-4	HL-60	DLD-1	LoVo	DU-145
**1**	6.292	2.111	2.117	0.967	0.563	NA ^b^
Doxorubicin ^a^	0.171	0.001	0.048	2.322	0.959	0.005

^a^ Doxorubicin was used as positive control; ^b^ NA = not active at 20 μg/mL.

**Table 3:** Inhibitory effects of diterpenoid **1** on the generation of superoxide anion and the release of elastase by human neutrophils in response to FMLP/CB.

Compounds	Superoxide anion		Elastase release
	IC_50_ (μg/mL)	Inh% ^a^	IC_50_ (μg/mL)
**1**	>10	20.55 ± 5.18	0.38 ± 0.14
DPI ^b^	0.80 ± 0.21		
Elastatinal ^b^			31.95 ± 5.92

^a^ Percentage of inhibition (Inh%) at a concentration of 10 μg/mL; ^b^ DPI (diphenylene indonium) and elastatinal were used as positive control.

## 3. Experimental Section

### 3.1. General Experimental Procedures

Optical rotations were measured on a Jasco P-1010 digital polarimeter. Infrared spectra were recorded on a Varian Diglab FTS 1000 FT-IR spectrometer; peaks are reported in cm^−1^. NMR spectra were recorded on a Varian Inova 500 or a Varian Mercury Plus 400 NMR spectrometers using the residual CHCl_3_ signal (*δ*_H_ 7.26 ppm) as internal standard for ^1^H NMR and CDCl_3_ (*δ*_C_ 77.1 ppm) for ^13^C NMR. Coupling constants (*J*) are given in Hz. ESIMS and HRESIMS were recorded by a Bruker APEX II mass spectrometer. Column chromatography was performed on silica gel (230–400 mesh, Merck, Darmstadt, Germany). TLC was carried out on precoated Kieselgel 60 F_254_ (0.25 mm, Merck); spots were visualized by spraying with 10% H_2_SO_4_ solution followed by heating. HPLC was performed using a system comprised of a Hitachi L-7100 pump, a Hitachi L-7455 photodiode array detector and a Rheodyne injection port. A normal phase column (Hibar 250 × 10 mm, Merck, silica gel 60, 5 μm) was used for HPLC.

### 3.2. Animal Material

Specimens of the gorgonian coral *Echinomuricea* sp. were collected by hand using scuba equipment off the coast of the southern Taiwan and stored in a freezer until extraction. This organism was identified by comparison with previous descriptions [8,9]. A voucher specimen (NMMBA-TW-GC-125) was deposited in the National Museum of Marine Biology and Aquarium, Taiwan. 

### 3.3. Extraction and Isolation

The freeze-dried and minced material of *Echinomuricea* sp. (wet weight 1.68 kg, dry weight 428 g) was extracted with a mixture of methanol (MeOH) and dichloromethane (CH_2_Cl_2_) (1:1). The residue was partitioned with ethyl acetate (EtOAc) and H_2_O. The EtOAc layer was partitioned between MeOH and *n*-hexane. The *n*-hexane layer was separated by silica gel and eluted using *n*-hexane/EtOAc/MeOH to yield 21 fractions A–U. Fraction M was separated on silica gel and eluted using a mixture of *n*-hexane/EtOAc to yield 10 fractions M1–M10. Fraction M3 was further purified by normal phase HPLC (*n*-hexane/acetone, 10:1) to yield compound **1** (33.7 mg).

Echinohalimane A (**1**): yellowish oil; [α]_25_^D^ −102 (*c* 1.69, CHCl_3_); IR (neat) ν_max_ 3375, 1755 cm^−1^; ^1^H (CDCl_3_, 400 MHz) and ^13^C (CDCl_3_, 100 MHz) NMR data, see Table 1; ESIMS: *m/z* 341 (M + Na)^+^; HRESIMS: *m/z* 341.2089 (calcd for C_15_H_18_O_4_ + Na, 341.2093).

### 3.4. Molecular Mechanics Calculations

Implementation of the MM2 force field [7] in CHEM3D PRO software from CambridgeSoft Corporation (Cambridge, MA, USA; ver. 9.0, 2005) was used to calculate molecular models.

### 3.5. Cytotoxic Assay

The cytotoxicity of diterepnoid **1** was assayed with a modification of the 3-(4,5-dimethylthiazol-2-yl)-2,5-diphenyltetrazolium bromide (MTT) colorimetric method according to previously described procedures [10,11]. 

### 3.6. Superoxide Anion Generation and Elastase Release by Human Neutrophils

Human neutrophils were obtained by means of dextran sedimentation and Ficoll centrifugation. Measurements of superoxide anion generation and elastase release were carried out according to previously described procedures [12,13]. Briefly, superoxide anion production was assayed by monitoring the superoxide dismutase-inhibitable reduction of ferricytochrome *c*. Elastase release experiments were performed using MeO-Suc-Ala-Ala-Pro-Valp-nitroanilide as the elastase substrate.

## 4. Conclusions

In general, halimane-type diterpenoids exist in terrestrial plants, and are rarely found in marine organisms [14]. The compounds of this type were found to possess a carbon skeleton intermediate between that of labdanes and clerodanes [15,16]. It is worth noting that echinohalimane A (**1**) is the first halimane-type derivative isolated from marine organisms belonging to phylum Cnidaria. The study material *Echinomuricea* sp. has begun to be transplanted in culturing tanks with a flow-through sea water system located in the National Museum of Marine Biology and Aquarium, Taiwan, for the extraction of additional natural products in order to establish a stable supply of bioactive material.
